# The relationship between thiol-disulfide balance and idiopathic sudden sensorineural hearing loss

**DOI:** 10.1016/j.bjorl.2021.01.004

**Published:** 2021-02-13

**Authors:** Yaser Said Çetin, Nazım Bozan, Koray Avcı, Mehmet Aslan, Özcan Erel

**Affiliations:** a Van Yüzüncü Yıl University, Faculty of Medicine, Department of Otorhinolaryngology, Van, Turkey; b Yüzüncü Yıl University, Faculty of Medicine, Department of Internal Medicine, Van, Turkey; c Ankara Yıldırım Beyazıt Univ. Med Fac. Biochem Dept. & Atatürk Research and Application Hospital, Ankara, Turkey

**Keywords:** Sudden sensorineural hearing loss, Oxidative stress, Total oxidant status, Total antioxidant status, Thiol/disulphide homeostasis

## Abstract

**Introduction:**

Impaired cochlear perfusion is a major etiological factor in idiopathic sudden sensorineural hearing loss. Oxidative stress has been shown to be a risk factor for oxidative damage.

**Objectives:**

We investigated the role of oxidative stress in idiopathic sudden sensorineural hearing loss by comparing serum levels of oxidant and antioxidant molecules including thiol/disulfide homeostasis paraoxonase, stimulated thiol/disulfide homeostasis paraoxonase, arylesterase, ceruloplasmin and myeloperoxidase in patients who did and did not recover after treatment.

**Methods:**

The amount of dynamic disulfide was calculated by determining half of the difference between the total thiols and native thiols. After the determination of native, total thiol, and disulfide amounts, the disulfide/total thiol percent ratio, native thiol/total thiol ratio and disulfide/native thiol percent ratio were calculated and then compared between the two groups. Additionally, clinical relationship between audiological recovery and native thiol, disulfide, disulfide/native thiol percent ratio, and disulfide/total thiol percent ratio levels was investigated. Blood samples were also analyzed for the assessment of thiol/disulfide homeostasis paraoxonase, stimulated thiol/disulfide homeostasis paraoxonase, arylesterase, ceruloplasmin, and myeloperoxidase levels.

**Results:**

A significant difference was found between the two groups with regard to total oxidant status disulfide, disulfide/native thiol percent ratio, disulfide/total thiol percent ratio, and native thiol/total thiol ratio levels (*p* =  0.001, *p* =  0.001, *p* =  0.001, *p* =  0.003, *p* =  0.001, *p* =  0.002, respectively). However, no significant difference was found between the two groups with regard to thiol/disulfide homeostasis paraoxonase, stimulated thiol/disulfide homeostasis paraoxonase, ceruloplasmin, and myeloperoxidase levels (*p* >  0.05 for all).

**Conclusion:**

The results supported the common hypothesis that vascular pathologies are the primary cause of idiopathic sudden sensorineural hearing loss and that other etiological factors ultimately result in vascular pathologies. The oxidant-antioxidant and thiol-disulfide balances were impaired in the idiopathic sudden sensorineural hearing loss group.

## Introduction

Idiopathic sudden sensorineural hearing loss (ISSNHL) is a syndrome characterized by rapid progression of a sensorineural hearing loss of 30 dB or more across at least three contiguous frequencies, occurring within a period of 72 h or less.^1^ The incidence of ISSNHL increases with age, ranging from 27 to 160 per 100,000 population. Although it is seen equally in both genders, its etiology remains unclear.^2^, ^3^ Moreover, its pathogenesis is multifactorial and various causes have been proposed, including viral infections, autoimmune ear disease, acoustic tumors, perilymphatic fistula, trauma, vascular disorders and psychogenic conditions. In 85% of the patients, the etiological diagnosis cannot be established by diagnostic tests and thus these patients are accepted as idiopathic. Spontaneous recovery may occur in 32%–65% of the patients.^4^, ^5^

Oxidative stress is defined as an imbalance between pro-oxidant and antioxidant molecules in the body, ultimately leading to the production of reactive oxygen species (ROS). It has also been shown that impaired microvascular perfusion during an ischemic event is associated with oxidative stress that leads to endothelial damage and affects terminal microvascular systems.^6^ In turn, ROS derivatives may lead to lipid peroxidation, thereby resulting in cellular metabolism disorder, nucleic acid damage and protein modification.^7^ The oxidation of the -SH (sulfhydryl) group in amino acids is an indicator of protein oxidation. Moreover, the oxidation of thiols, functional sulfhydryl groups, and thiol groups leads to the formation of disulfide bonds. When the oxidative stress decreases, these disulfide bonds are reduced to thiol groups, which can be an indicator of oxidative stress.^8^

In this study, we investigated the role of oxidative stress in ISSNHL by comparing serum levels of oxidant and antioxidant molecules including thiol/disulfide homeostasis (TDH), paraoxonase (PON), stimulated PON (SPON), arylesterase (ARES), and myeloperoxidase (MPO) in patients that did and did not recover after treatment.

## Methods

The study included ISSNHL patients who were diagnosed and followed in our clinic between 2017 and 2020. An age-matched control group of healthy volunteers was included in the study and was compared with the patient group. Patients detected with the established causes of ISSNHL (temporal bone fracture, trauma, vestibular schwannoma) were excluded from the study. Additionally, patients with acute or chronic ear infections, chronic systemic diseases (cancer, diabetes mellitus, chronic heart failure, and Parkinson’s or Alzheimer’s disease), autoimmune diseases, and acute or chronic infectious diseases, patients receiving antioxidant drug therapies (angiotensin-converting enzyme [ACE] inhibitors, beta blocker with antioxidant properties, or antioxidant vitamins), and patients with a history of smoking within the last four weeks were also excluded from the study. An informed consent was obtained from each participant and the study was approved by Van Training and Research Ethics Committee (Approval nº 08-2017, Date: October 26, 2017).

Following an 8 h fasting period, blood samples were taken from each participant to assess their serum oxidative stress levels. Samples were placed in biochemical tubes and centrifuged at 2300 rpm for 10 min and then stored at −80 °C until analysis. Reducible disulfide bonds were reduced to form free functional thiol groups. Formaldehyde was used to remove residual sodium monohydrate and DTNB (5,5′-dithiobis-[2-nitrobenzoic acid]) products. Subsequently, both reduced and native natural thiol groups were formed.

The amount of dynamic disulfide was calculated by determining half of the difference between the total thiols and native thiols. After the determination of native, total thiol, and disulfide amounts, the disulfide/total thiol percent ratio (D/TT), native thiol/total thiol ratio (NT/TT), and disulfide/native thiol percent ratio (D/NT) were calculated and then compared between the two groups.^9^ Additionally, clinical relationship between audiological recovery and native thiol, NT/TT, disulfide, D/NT, and D/TT levels was investigated. Blood samples were also analyzed for the assessment of PON, SPON, ARES, CLP, and MPO levels using previously described techniques.^10^, ^11^

### Audiometric examination and average hearing levels

A routine audiometric test was administered for each participant by an audiologist blinded to the study. Hearing thresholds were determined by pure tone audiometry (PTA) in a soundproof booth using an Interacoustics® audiometer (Assens, Denmark; Model: AC40). Audiometry was performed at frequencies from 250 Hz to 8000 Hz. Hearing thresholds were measured in dB HL units. Average hearing threshold at each frequency was determined for each ear of all participants and then these levels were compared between the two groups. The category of hearing loss was defined based on the PTA that was taken as the average threshold across 500, 1000, 2000, and 4000 Hz: mild hearing (26–40 dB), moderate hearing loss (41–55), moderate-severe hearing loss (56–70 dB), severe hearing loss (71–90), and very severe hearing loss (91 dB or higher). The response of the patients to the treatment was classified as follows: 1) Completely recovered: the posttreatment hearing loss value less than 10 dB compared to the initial hearing loss value or the intact ear PTA; 2) Partially recovered: posttreatment recovery of more than 50% when compared to the intact ear PTA; and 3) Unrecovered: a recovery of less than 50% compared to the intact ear PTA.

### Statistical analysis

Data were analyzed using SPSS for Windows version 24.0 (IBM Corp. Released 2016, Armonk, NY: IBM Corp.). Normal distribution of continuous variables was determined using Shapiro-Wilk and Skewness-Kurtosis tests and parametric tests were used for the analyses since the variables were found to have a normal distribution. Continuous variables were expressed as mean, Standard Deviation (SD), minimum, and maximum. Categorical variables were expressed as frequencies (n) and percentages (%). Continuous variables were compared using Independent *t*-test. Correlations were determined using Pearson’s Correlation Coefficient. A *p*-value of < 0.05 was considered significant.

## Results

A total of 43 ISSNHL patients and 43 control subjects that were otherwise healthy were included in the study. The patient group was comprised of 36 (83.7%) women and 7 (16.3%) men with a mean age of 39.5 ± 10.3 years and the control group comprised 30 (69.8%) women and 13 (30.2%) men with a mean age of 38.7 ± 9.5 years. No significant difference was found between the two groups with regard to age and gender distribution (*p* = 0.54).

In the patient group, a pretreatment ISSNHL of 30–50 dB was detected in 11 (25.6%), 51–70 dB in 14 (32.6%), and 71–90 dB in 23 (53.5%) patients. In the same group, median symptom duration was 7 (range, 1–30) days. High-frequency ISSNHL (descending type) was present in 28 (65.1%), low-frequency ISSNHL (ascending type) was present in 11 (25.6%), and an ISSNHL affecting all frequencies (flat type) was present in 4 (9.3%) ears. In terms of treatment response, remission was detected in 23 (53.5%), partial recovery was detected in 15 (34.9%), and no recovery was detected in 5 (11.6%) patients.

A significant difference was found between the two groups with regard to total oxidant status (TOS), disulfide, D/NT, D/TT, and NT/TT levels (*p* =  0.001, *p* =  0.001, *p* =  0.001, *p* =  0.003, *p* =  0.001, *p* =  0.002, respectively). However, no significant difference was found between the two groups with regard to PON, SPON, CLP, and MPO levels (*p* >  0.05 for all) (Table 1).

**Table 1:** Comparison of results in Idiopathic Sudden Sensorineural Hearing Loss (ISSNHL) patients and control group.

	ISSNHL	Control	*p*-value
Total antioxidant capacity	11.2 ± 10.8	1.6 ± 0.2	< 0.001
Total oxidant status	11.2 ± 10.3	8.6 ± 9.7	0.244
Paraoxoanase-1	176 ± 115	181 ± 150	0.871
Stimulated paraoxoanase	594 ± 423	604 ± 526	0.921
Arylesterase	276 ± 46	318 ± 53	< 0.001
Ceruloplasmin	72 ± 28	69 ± 47	0.651
Myeloperoxidase	94 ± 49	111 ± 53	0.142
Native thiol (SH)	356 ± 65	424 ± 48	< 0.001
Total thiol (SH + SS)	395 ± 65	460 ± 46	< 0.001
Disulphide (SS)	20 ± 5	18 ± 5	0.118
SS/SH (%)	6 ± 3	4.3 ± 1.6	0.003
SS/Total SH (%)	5.2 ± 2.1	3.9 ± 1.3	0.001
SH/Total SH (%)	89.6 ± 4.3	92.1 ± 2.6	< 0.002

ISSNHL, Idiopathic Sudden Sensorineural Hearing Loss; SH, Native Thiol; SS, Disulphide.

Data is expressed either as mean ± standard deviation (range: minimum–maximum) or median-interquartile range (range: minimum–maximum), *p* <  0.05 value was accepted as significant level and the significant differences between the groups are shown in bold.

In the correlation analysis, only PON, among all the oxidative stress parameters analyzed in the study, established a significant correlation with the severity of pretreatment ISSNHL (*p* =  0.038) (Table 2).

**Table 2:** Correlation between serum antioxidant level and air-conduction PTA of the ISSNHL.

	The relationship between pre-treatment pure tone mean level and oxidative stress markers	*p*^a^	The relationship between post-treatment pure tone mean level and oxidative stress markers	*p*^a^
Total antioxidant capacity TAC	−0.038	0.808	−0.102	0.515
Total oxidant status TOS	0.067	0.672	−0.010	0.947
Paraoxoanase-1 PNX	0.317^a^	0.038^a^	0.016	0.918
Stimulated paraoxoanase	0.271	0.079	−0.013	0.934
Arylesterase	−0.001	0.997	0.240	0.122
Ceruloplasmin	−0.127	0.422	−0.059	0.711
Myeloperoxidase	−0.154	0.324	0.043	0.787
Native thiol	−0.046	0.768	0.146	0.352
Total thiol	−0.080	0.611	0.148	0.342
Disulphide	−0.169	0.279	0.009	0.953

a Significance values according to Pearson Correlation Coefficient.

## Discussion

The etiology and ideal treatment approach of ISSNHL remains contradictory. Although most of the patients have an idiopathic form of ISSNHL, vascular disorders have been reported as the most common cause of the disease.^12^ Recently, there have been numerous studies investigating the role of ROS in hearing loss. However, the common question is whether ROS lead to hearing loss or ROS are caused by the necrosis or apoptosis in auditory cells. Previous studies indicated that reoxygenation or reperfusion that will develop after ischemia due to vascular disorders leads to tissue damage in proportion to the duration of exposure to ischemia. This situation, which is called the oxygen paradox, is considered to be caused by free radicals that are formed as a result of reperfusion.^13^ Adequate oxygenation and circulation are required for the inner ear to function properly and a disturbance in the circulation of the inner ear leads to vascular endothelial damage.^14^ It has been suggested that the increased production of highly oxidant species such as nitric oxide and peroxynitrite and proinflammatory cytokines may lead to endothelial damage, thereby resulting in sudden hearing loss.^15^ Additionally, increased levels of oxidative stress molecules have been shown in patients with sudden hearing loss.^16^ Under normal conditions, increased ROS production stimulates the antioxidant systems. Antioxidant molecules play an important role in detoxification, cell regeneration, regulation of the cellular enzymatic activity, and apoptosis. Moreover, they also protect the body against the adverse effects of ROS. Insufficiency of the antioxidant system leads to oxidative stress through the effects of oxidant molecules, and the antioxidant system has been shown to cause auditory neuropathy.^17^ A previous study indicated that ROS byproducts contribute to the progressive degeneration of neural morphology in cranial nerves and cause abnormal positioning of ROS in actin filaments.^18^

Some of the antioxidants that try to prevent the damage caused by free radicals are enzymes and some of them are non-enzymes molecules. Although the antioxidant/oxidant status of the body and the concentration of antioxidant/oxidant molecules can be assessed separately, the overall antioxidant/oxidant status can be assessed more easily by the measurement of total antioxidant status (TAS) and TOS. In our study, the TOS levels were higher in the patient group compared to the control group, which indicates that a cellular defense is formed against oxidative stress. PON and ARES are enzymes in the esterase group that are encoded by the same gene and have similar active centers. In our study, no significant difference was found with regard to serum levels of PON and SPON, both of which are lipid metabolism products. Serum PON is an enzyme associated high-density lipoprotein (HDL) and exhibits anti-atherosclerotic activity by inhibiting low-density lipoprotein (LDL) oxidation. Significantly elevated PON levels are mostly seen in the presence of atherosclerotic lesions.^19^ For these reasons, it can be assumed that there can be no relationship between atherosclerosis and ISSNHL and that ARES can detoxify organophsophates such as PON.

Ceruloplasmin is a key protein that is responsible for 90% of copper transport in plasma and is synthesized predominantly in the liver. This protein protects tissues against the adverse effects of iron-containing ROS and also exhibits antioxidant and cell protective activity. MPO is a lysosomal enzyme secreted by leukocytes in response to oxidative stress. Increased MPO activity, which leads to nitric oxide inactivation and thereby reduces the vasodilatory and anti-inflammatory effects of nitric oxide, has been blamed in numerous pathological mechanisms, predominantly including vascular pathologies. In our study, the significantly reduced serum ARES level in the patient group and the absence of a significant difference between the two groups with regard to serum levels of other antioxidants (such as PON, MPO, ceruloplasmin) suggest that different enzymes may play a role in the antioxidant system.

Thiols are composed of hydrogen and sulfur molecules bound to a single carbon atom and play a major role in the neutralization of body oxidants. Disulfide bonds are attached to thiol groups to reduce the thiol-disulfide balance. Plasma proteins, particularly albumin, are highly susceptible to oxidation since the thiol group in their structure contains free sulfhydryl. In the presence of oxidative stress, free sulfhydryl groups between two proteins form disulfide bonds through their antioxidant properties. The thiol-disulfide balance, on the other hand, is a marker indicating the oxidation process of proteins.^20^ Reduced thiol levels have been shown in numerous diseases including diabetes mellitus, kidney disease, alcoholic liver cirrhosis, chronic kidney failure, cardiovascular diseases, neurological disorders, and cancer.^21^ Additionally, reduced thiol levels have been shown in patients with sudden hearing loss as well.^22^ On the other hand, cell membranes can also be damaged by free radicals, which results from the “stealing” of electrons from the lipids by free radicals during lipid peroxidation. Malondialdehyde (MDA) and thiobarbituric acid reactive substances (TBARS) are common ways to measure lipid peroxidation products in cells, tissues, and body fluids. Elias et al.^23^ evaluated 80 SSNHL patients and divided the patients into three groups based on the recovery of hearing loss (total recovery, partial recovery, and no recovery). The authors evaluated the relationship between comorbidities or serum concentration of TBARS and recovery of hearing loss and found no significant correlation between the concentration of TBARS and the severity of the initial hearing loss or the prognosis of hearing recovery. However, the authors found a significant relationship between the presence of diabetes and dyslipidemia and worse hearing prognosis in patients with SSNHL.^23^ In our study, serum levels of oxidative stress parameters including TOS, ARES, native thiol, and total thiol were significantly lower in the patient group compared to the control group. Of note, these reduced thiol levels indicate that antioxidant molecules are activated by sudden hearing loss and that the antioxidant system plays a protective role against cell metabolism. On the other hand, although serum levels of oxidative stress markers were increased in our recovered, partially recovered, and unrecovered patients, no significant correlation was found among these groups with regard to recovery.

Previous studies indicated that noise-induced hearing loss can be controlled by exogenous antioxidants. To date, various antioxidants have been tried in the treatment of ISSNHL and there have been several studies reporting on beneficial effects of antioxidants on inner ear damage and ISSNHL. However, although antioxidants such as N-acetylcysteine, vitamin C and E, selenium, ginkgo biloba extracts, and fish oil have been tried for such treatments, their effects have not yet been elucidated.^24^ Therefore, the role of antioxidant therapies in the treatment of ISSNHL remains contradictory. On the other hand, although antioxidants including enzymatic radical scavengers (such as superoxide and hydrogen peroxide scavengers), iron chelators (desferrioxamine), and xanthine oxidase inhibitors (allopurinol, oxypurinol) represent the only solution for providing protection against reperfusion injury, antioxidant therapy is not recommended in the treatment of ISSNHL,^25^ which could be attributed to the scarcity of randomized controlled studies conducted with antioxidants. Some previous studies showed that antioxidants, ROS, and, in particular, the thiol-disulfide and thiol/disulfide homeostasis were elevated in patients with sudden hearing loss.^22^, ^26^

Combined use of antioxidants and steroids in ISSNHL treatment may have a synergistic effect and thus may be complementary in improving hearing. Further large-scale randomized blinded studies are needed to explore whether early addition of antioxidants to standard steroid therapy would improve recovery of ISSNHL, particularly in high-frequency hearing losses that are less responsive to treatment.^27^ On the other hand, nano-antioxidant molecules have been shown to protect the central nervous system against ROS.^28^ Nevertheless, despite the great promise and potential of these molecules, their application in medicine is still limited due to their low bioavailability. Additionally, they are easily degraded and thus their antioxidant capacities can decrease significantly.^29^

## Conclusion

The present study obtained similar findings to those of other studies that confirmed the role of oxidative stress in the etiopathogenesis of ISSNHL. Of note, the total thiol, D/NT, and D/TT values were significantly higher in the ISSNHL group compared to the control group. However, the oxidant-antioxidant and thiol-disulfide balances were impaired in the ISSNHL group and no significant correlation was found between this imbalance and the severity of hearing loss or treatment response. The assessment of serum levels of oxidative parameters in ISSNHL patients can be helpful in organizing the treatment protocol. However, to make definitive assumptions on this subject, further large-scale studies performing correlation analyses are needed.

## Ethical approval

All procedures performed in this study were in accordance with the ethical standards of the institutional committee and with the 1964 Helsinki Declaration and its later amendments or comparable ethical standards.

## Conflicts of interest

The authors declare no conflicts of interest.
